# A potential mechanism underlying atypical antipsychotics-induced lipid disturbances

**DOI:** 10.1038/tp.2015.161

**Published:** 2015-10-20

**Authors:** H L Cai, Q Y Tan, P Jiang, R L Dang, Y Xue, M M Tang, P Xu, Y Deng, H D Li, J K Yao

**Affiliations:** 1 Department of Pharmacy, The Second Xiangya Hospital of Central South University, Changsha, China; 2 The Institute of Clinical Pharmacy, Central South University, Changsha, China; 3 Department of Pharmaceutical Sciences, University of Pittsburgh School of Pharmacy, Pittsburgh, PA, USA; 4 Medical Research Service, VA Pittsburgh Healthcare System, University of Pittsburgh, Pittsburgh, PA, USA; 5 School of Pharmacy, Guilin Medical University, Guilin, China; 6 School of Pharmacy, Central South University, Changsha, China; 7 Department of Psychiatry, University of Pittsburgh School of Medicine, Pittsburgh, PA, USA

## Abstract

Previous findings suggested that a four-protein complex, including sterol-regulatory element-binding protein (SREBP), SREBP-cleavage-activating protein (SCAP), insulin-induced gene (INSIG) and progesterone receptor membrane component 1 (PGRMC1), within the endoplasmic reticulum appears to be an important regulator responsible for atypical antipsychotic drug (AAPD)-induced lipid disturbances. In the present study, effects of typical antipsychotic drug and AAPDs as well as treatment outcome of steroid antagonist mifepristone (MIF) on the PGRMC1/INSIG/SCAP/SREBP pathway were investigated in rat liver using real-time quantitative polymerase chain reaction (qPCR) and western blot analysis. In addition, serum triacylglycerol, total cholesterol, free fatty acids and various hormones including progesterone, corticosterone and insulin were measured simultaneously. Following treatment with clozapine or risperidone, both lipogenesis and cholesterogenesis were enhanced via inhibition of PGRMC1/INSIG-2 and activation of SCAP/SREBP expressions. Such metabolic disturbances, however, were not demonstrated in rats treated with aripiprazole (ARI) or haloperidol (HAL). Moreover, the add-on treatment of MIF was effective in reversing the AAPD-induced lipid disturbances by upregulating the expression of PGRMC1/INSIG-2 and subsequent downregulation of SCAP/SREBP. Taken together, our findings suggest that disturbances in lipid metabolism can occur at an early stage of AAPD treatment before the presence of weight gain. Such metabolic defects can be modified by an add-on treatment of steroid antagonist MIF enhancing the PGRMC1 pathway. Thus, it is likely that PGRMC1/INSIG-2 signaling may be a therapeutic target for AAPD-induced weight gain.

## Introduction

High rates of comorbidity with metabolic disturbances have been associated with schizophrenia (SZ) patients following treatment with atypical antipsychotic drugs (AAPDs).^1^ The review by De Hert *et al.*^2^ found that the prevalence rate of the metabolic syndrome (MetS) was two to three times higher in patients with SZ or schizoaffective disorder than in the general population. Moreover, first-episode SZ patients who started with AAPDs had a three times higher incidence rate of MetS as compared with the first-episode SZ patients treated with typical antipsychotics.^1^ Such MetS^3^ consisting of high blood pressure, hyperglycemia, insulin resistance, weight gain and dyslipidemia may contribute to the increased cardiovascular mortality in this debilitating disease. Therefore, research efforts aimed at mitigating these AAPD-induced metabolic disturbances are of paramount importance.

The mechanisms underlying the influences of AAPDs on specific neuroendocrine and metabolic dysfunctions remain poorly understood. Previous studies have linked the effects of serotonin 5-HT_2_ (ref. 4) and histamine H_1_ receptors^5^ to AAPD-induced weight gain, possibly resulting from an increased dietary intake. Recent studies, however, suggested that the sterol-regulatory element-binding proteins (SREBPs) are central in the allosteric control of a variety of lipid biosynthetic pathways.^6^ Specifically, SREBP-1 is primarily responsible for regulation of fatty acid and triacylglycerol metabolism, whereas SREBP-2 is the main regulator for cholesterol metabolism.^6^ In the liver, excess SREBP activity could account for elevated circulating cholesterol, free fatty acids (FFAs) and triacylglycerols.^6^

**Figure 1:**
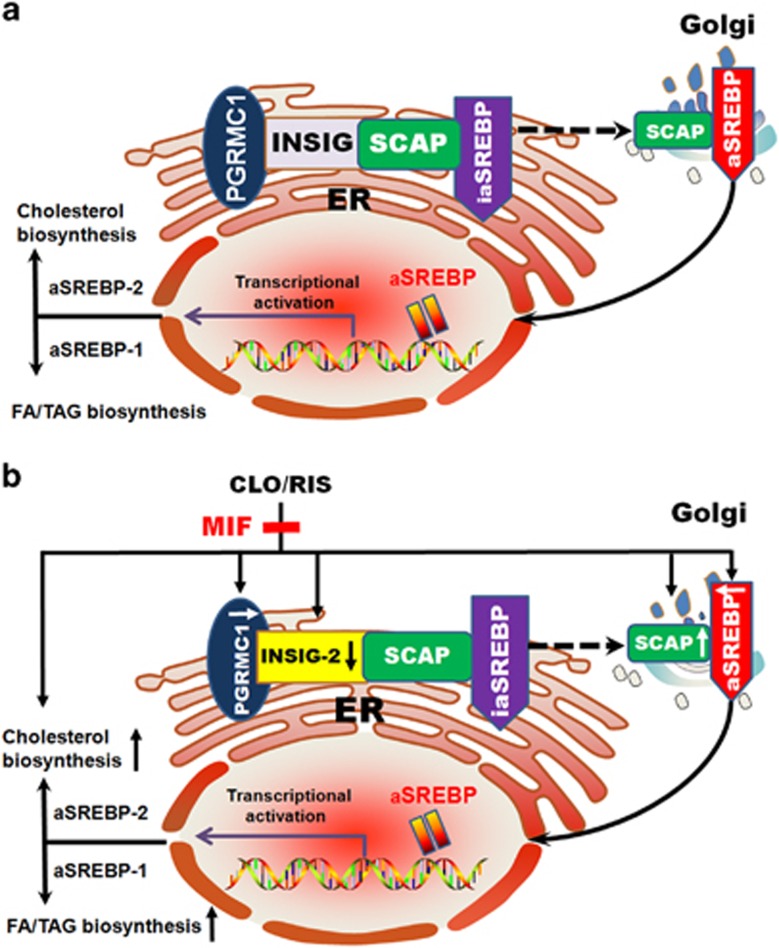
Schematic diagrams illustrating the mechanism of (**a**) the involvement of PGRMC1/INSIG/SCAP/SREBP signaling in the lipid biosynthesis. (**b**) AAPD-induced hepatic inhibition of PGRMC1/INSIG-2 and subsequent activation of SCAP/SREBP that contributes to the increased biosynthesis of lipids in the liver. The add-on MIF treatment can reverse AAPD-induced metabolic disturbances by upregulating the expression of PGRMC1/INSIG-2 followed by downregulation of SCAP/SREBP. AAPD, atypical antipsychotic drug; aSREBP, active sterol-regulatory element-binding protein; ER, endoplasmic reticulum; FA, fatty acid; iaSREBP, inactive sterol-regulatory element-binding protein; INSIG, insulin-induced gene; MIF, mifepristone; PGRMC1, progesterone receptor membrane component 1; SCAP, SREBP-cleavage activating protein; TAG, triacylglycerol.

SREBPs form complexes with SREBP-cleavage-activating protein (SCAP), a transport protein that escorts inactivated SREBPs (ia-SREBPs) from the endoplasmic reticulum (ER) to Golgi apparatus, where ia-SREBPs are activated before entering in nuclear and regulating genes for lipid biosynthesis (Figure 1a). Insulin-induced genes (INSIGs) are then bound to SCAP to promote the ER retention of SCAP and block the ER-to-Golgi transportation of SCAP/SREBP when cholesterol is elevated, and thus to reduce lipogenesis and cholesterogenesis.^7^ INSIGs have two isoforms consisting of INSIG-1, a target of nuclear SREBPs and their gene rises and falls coordinately with nuclear SREBP levels, and INSIG-2, expressed at a low but constant level and negatively regulated by insulin without being influenced by SREBPs.^8^ *INSIG-2* gene polymorphisms and gene interactions between *INSIG-1* and *INSIG-2* had been linked to the adverse effects of weight gain and the MetS in SZ patients following AAPD treatment.^9, 10^ It is surmized that INSIGs may be candidate genes for AAPD-induced lipid disturbances.

The biochemical pathways involving SREBP may be altered by a variety of APPDs. Olanzapine, clozapine (CLO) and risperidone (RIS) can elicit significant upregulation of SREBP-1 and SREBP-2 and their downstream target genes leading to increased lipid and cholesterol synthesis.^11, 12, 13^ Even a single intraperitoneal injection of CLO or olanzapine can induce elevation of serum FFA, followed by hepatic accumulation of lipids.^14, 15^ On the other hand, quetiapine and aripiprazole (ARI) are virtually devoid of any metabolic side effects.^11, 12, 13^

As illustrated in Figure 1a, INSIG and SCAP are also bound to progesterone receptor membrane component 1 (PGRMC1),^16^ which have regulatory function in the lipid biosynthesis.^17, 18^ PGRMC1 is considered as a member of a multiprotein complex that preferentially binds to various steroids in the physiological condition,^17^ exerting a variety of biological functions such as sterol synthesis, damage repair, drug and hormone metabolism, apoptosis suppression and cholesterol regulation.^18^ PGRMC1 is highly expressed in the rodent and human liver, and mostly located in the ER.^17, 18^ It is likely that the PGRMC1-associated progesterone-binding activity has a similar affinity for corticosterone, testosterone and cortisol.^19^ Progesterone may have a regulatory role in the suppression of PGRMC1 expression.^20^

Taken together, we hypothesized that PGRMC1/INSIG might be involved in AAPD-induced metabolic disturbances in the liver. The primary aims of the present study were to test (a) whether the hepatic PGRMC1/INSIG/SCAP/SREBP pathway underlies the mechanisms of AAPD-induced lipid disturbances and (b) whether the steroid antagonist mifepristone (MIF)^21, 22, 23^ can modify the AAPD-induced lipid disturbances by its ability of restoring the deficits in the PGRMC1/INSIG/SCAP/SREBP pathway.

## Materials and methods

### Chemicals

CLO, RIS, ARI, haloperidol (HAL), sertraline (SER) and MIF were purchased from Eastbang Pharmaceuticals (Guangzhou, China). The antibodies of PGRMC1 and INSIG-1 were purchased from Proteintech (Chicago, IL, USA), whereas INSIG-2, SCAP, SREBP-1 and SREBP-2 were obtained from Santa Cruz Biotechnology (Santa Cruz, CA, USA). TRIzol reagent was purchased from Life Technologies (Gaithersburg, MD, USA). The internal standard β-actin antibody was purchased from Proteintech. In addition, the enzyme-linked immunosorbent assay kits for progesterone, corticosterone and insulin assays were purchased from Antibodies-online (Shanghai, China).

### Preparation of drug solution

In the presence of insolubility, CLO (4.2 mg ml^−1^), RIS (0.2 mg ml^−1^) and ARI (0.4 mg ml^−1^) were initially dissolved in 0.5% (v/v) of acetic acid and re-suspended into 10% (v/v) of 0.9% saline solution containing 0.5% Tween 80. Then, the final concentration was adjusted with additional 0.9% saline solution containing 0.5% Tween 80, and the pH was adjusted between 5.6 and 6.0 using 0.1 M NaOH solution. HAL (0.2 mg ml^−1^) and SER (1.4 mg ml^−1^) were directly dissolved in 0.9% saline containing 0.5% Tween 80. MIF (40 mg ml^−1^) was prepared as a suspension using 0.2% Tween 80 dissolved in sterile water containing 0.2% carboxymethylcellulose.

### Animals

To avoid progester–one fluctuation during the menstrual cycle, only male Sprague–Dawley rats were used in the present study. The Sprague–Dawley rats weighing 150–200 g were purchased from the Second Xiangya Hospital. All rats were maintained at 22–25 °C and in humidity 50–60% under a 12-h light/dark cycle with free access to commercial rat chow (SLAC Laboratory Animal, Shanghai, China) and water. They were acclimatized for one week before experimentation. Each rat was housed in a separate cage and recorded with body weight and food intake daily. The drug doses were adjusted to the weight changes daily. The research protocol using animals was approved by the local Ethics Committee of Central South University. All the experimental procedures conformed to the Declaration of Helsinki and the Regulations of Experimental Animal Administration, which were issued by the State Committee of Science and Technology of the People's Republic of China.

### Effects of antipsychotics on the hepatic PGRMC1/INSIG/SCAP/SREBP pathway

In the first series of experiments (Supplementary Table 1), 42 rats were randomly divided into six groups (*n*=7) treated with different antipsychotic drugs: (1) normal control (NC), (2) SER, (3) HAL, (4) ARI, (5) RIS and (6) CLO. They were administered intraperitoneal injection of vehicle (0.9% saline containing 0.5% Tween 80), SER (7 mg kg^−1^ per day), HAL (1 mg kg^−1^ per day), ARI (2 mg kg^−1^ per day), RIS (1 mg kg^−1^ per day) or CLO (21 mg kg^−1^ per day) at baseline and daily for another 4 weeks. The doses for the antipsychotics used in rats were converted from clinically prescribed dosages using the body surface area normalization method.^24^ Their human-equivalent dosages for a 60-kg person were 70 mg/day for SER,^25^ 10 mg/day for HAL,^26^ 20 mg/day for ARI,^27^ 10 mg/day for RIS^28^ and 200 mg/day for CLO.^29^ As rodents have significantly shorter half-life of antipsychotics as compared with humans,^14^ we intended to use relatively high drug doses to challenge the SREBP system and to compensate the rapid clearance of AAPDs in rats, with the aim of detecting marked transcriptional responses. HAL and RIS were selected at the upper end dosing, whereas ARI was chosen at a medium dose because evidence shows that doses over 20 mg/day provide no additional benefits.^27^ In our preliminary experiment, we selected 450 mg/day CLO (equals to 46 mg kg^−1^ per day for rats) and found that the rats could not tolerate such a high dose in the 4-week study time frame. Therefore, we finally chose a low maintenance dose of 200-mg/day CLO to ensure the survival rate of the CLO group. The rats were fasted for 12 h before being killed. Samples were taken from the liver median lobe and freeze-clamped immediately in liquid nitrogen before storage at −80 °C. Truncal blood was collected in vacuum tubes. The serum was separated from whole blood by centrifugation at 3600 *g* for 7 min at 4 °C and was stored under −80 °C before laboratory assays.

### Effects of the add-on MIF treatment on the hepatic PGRMC1/INSIG/SCAP/SREBP pathway

In the second series of experiments (Supplementary Table 1), 42 rats were randomly assigned to one of the following six groups (*n*=7 each): (1) NC (vehicle), (2) MIF (alone), (3) CLO, (4) CLO+MIF, (5) RIS and (6) RIS+MIF.

The CLO (3) and RIS (4) groups were first administered alone intraperitoneal injection of the same doses as described in the first series of experiments. In the add-on experiments (four and six groups), MIF (200 mg kg^−1^ per day) was administrated by oral gavage daily.^21^ The NC vehicle group received both intraperitoneal injection of 0.9% saline containing 0.5% Tween 80 and oral gavage of 0.2% carboxymethylcellulose and 0.2% Tween 80 dissolved in sterile water. The experimental duration and procedures of sampling were identical to the first series of experiments.

### Real-time qPCR

For real-time quantitative polymerase chain reaction (qPCR) analysis, total RNA from the liver tissue was isolated using the TRIzol reagent following the manufacturer's protocols.^30^ After confirmation of purity by spectrophotometry, quantification of mRNAs was carried out on a Bio-rad Cx96 Detection System (Hercules, CA, USA) using the SYBR green PCR kit (Applied Biosystems, Foster City, CA, USA) and gene-specific primers. Complementary DNA (cDNA; 5 ng) sample was used with 40 cycles of amplification and each cDNA was tested in triplicate. Relative quantitation for PCR product was normalized using internal standard β-actin. The sequences of gene-specific primers were summarized in Supplementary Table 2.

### Western blot analyses

For western blot experiments, total protein was prepared from 100 mg of tissue and the concentration was measured using the Bradford method.^31^ Approximately 50 μg protein was loaded onto a precast 12% sodium dodecylsulphate-polyacrylamide gel electrophoresis gel. All the antibodies were properly diluted (PGRMC1, 1:1500; INSIG-1, 1:1000; INSIG-2, 1:200; SCAP, 1:200; SREBP-1, 1:200; and SREBP-2, 1:500) before use. All membranes were subsequently probed with a 1:4000 dilution of β-actin antibody. The film signal was digitally scanned and then quantified using the Image J software (National Institutes of Health, Bethesda, MD, USA). The signals were normalized using β-actin as the internal standard.

### Biochemical assays

Serum levels of progesterone, corticosterone and insulin were determined in duplicate using commercial enzyme-linked immunosorbent assay kits. Serum triacylglycerols, total cholesterol and FFAs were measured using Architech C8000 Automatic Biochemical Analyzer (Abbott, Wiesbaden, Germany) in the clinical laboratory at the Second Xiangya Hospital.

### Statistical analysis

Data were expressed as the means and s.d., and were analyzed using the SPSS 19.0 software package (SPSS, Chicago, IL, USA). Changes in body weight and food intake were compared across groups using repeated-measures analysis of variance models with Dunnett's *t*-test^21^ for *post hoc* comparisons. These models included treatment group as a between-subject factor and time as a within-subject factor. Owing to small samples and non-normally distributed variance, differences in relative mRNA and protein expression as well as serum biochemical parameters across groups at the end point were determined using nonparametric Kruskal–Wallis one-way analysis of variance^32^ followed by pairwise multiple comparisons. The significance level was established at *P*<0.05.

## Results

### Alterations of weight gain and food intake after AAPDs or add-on MIF treatment

Both body weight and dietary intake were recorded daily following 4-week treatment of various antipsychotic drugs as well as add-on MIF treatment. The weekly average percentage of weight gain and accumulated food intake were shown in Supplementary Figure 1a–d, respectively. A significant lower percent of weight gain was found in the CLO group after 2- and 28-day treatment than in those of NC groups. Similarly, significant reductions of accumulated food intake were also found in these CLO-treated rats. There were no significant differences in either weight gain or dietary intake among other treated groups (Supplementary Figure 1a and b).

Following 4 weeks of add-on MIF treatment, neither weight gain (Supplementary Figure 1c) nor dietary intake (Supplementary Figure 1d) were significantly altered by the steroid antagonist MIF.

### Effects of antipsychotic drugs on serum metabolic parameters

Serum major lipids and hormone levels were determined following 4-week treatment with AAPDs as compared with NCs and SER group (antidepressant). Both CLO and RIS groups had significant increases in serum triacylglycerols, total cholesterol, FFAs, progesterone and corticosterone levels than those of the NC group (Table 1). On the other hand, there were no significant changes of these serum parameters between NC and HAL (or ARI) groups. Similarly, no significant changes were noted between NC and SER groups. In addition, levels of serum insulin remained essentially the same among all groups.

### Effects of add-on MIF treatment on serum metabolic parameters

There were no significant changes of both serum lipid and hormone parameters between NC and MIF-treatment-alone groups (Table 1). In accordance with our findings above, major serum metabolic parameters were significantly elevated in both CLO and RIS groups. Following add-on treatment with MIF, however, the major lipid parameters were drastically reduced. Interestingly, serum progesterone and corticosterone remained at elevated levels. Again, there were no significant changes of fasting insulin levels among all groups.

### Differential modulatory effects of antipsychotics on the hepatic PGRMC1/INSIG/SCAP/SREBP pathways

**Figure 2:**
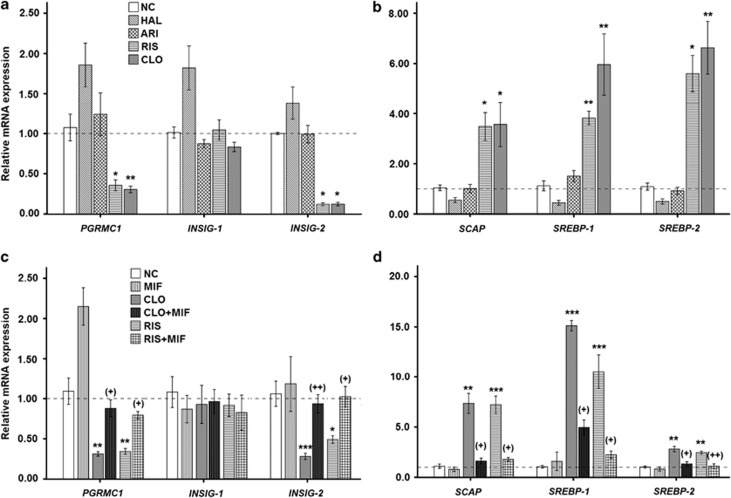
Hepatic expression of *PGRMC1/INSIG/SCAP/SREBP* mRNA in rats after 4-week treatment with different antipsychotic drugs (**a,** **b**); add-on MIF medication with AAPD monotherapy (**c**, **d**) using real-time qPCR. *P<0.05, **P<0.01 and ***P<0.0001 as compared with controls. ^+^*P*<0.05 and ^++^*P*<0.01 in parentheses as compared with corresponding AAPD monotherapy group. Relative expression values were presented as a normalized ratio to the *β-actin* mRNA level. AAPD, atypical antipsychotic drug; ARI, aripiprazole; CLO, clozapine; CLO+MIF, clozapine with add-on MIF treatment; HAL, haloperidol; MIF, mifepristone; NC, normal control; qPCR, quantitative polymerase chain reaction; RIS, risperidone; RIS+MIF, risperidone with add-on MIF treatment.

To explore the potential modulatory effects of various antipsychotics used for SZ on the PGRMC1/INSIG/SCAP/SREBP pathway, hepatic gene (Figure 2a and b) of the six key factors (that is, *PGRMC1, INSIG-1, INSIG-2, SCAP, SREBP-1* and *SREBP-2*) were compared after 4-week antipsychotics treatment.

In CLO and RIS groups, the expressions of *PGRMC1* and *INSIG-2* were significantly reduced without affecting *INSIG-1* (Figure 2a), whereas *SCAP/SREBP* expressions were markedly elevated (Figure 2b) as compared with the NC group. On the other hand, there were no significant changes of relative mRNA expressions of six tested factors between NC and HAL (or ARI) groups.

**Figure 3:**
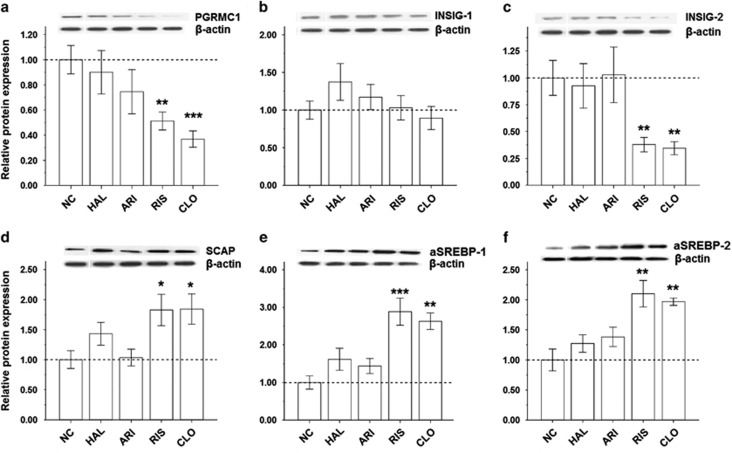
Quantification of the PGRMC1/INSIG/SCAP/SREBP protein in the rat liver by western blotting following 4-week treatment of various antipsychotic drugs (HAL, ARI, RIS and CLO): (**a**) PGRMC1, (**b**) INSIG-1, (**c**) INSIG-2, (**d**) SCAP, (**e**) SREBP-1 and (**f**) SREBP-2. *P<0.05, **P<0.01 and ***P<0.0001 as compared with controls. Relative expression data were calculated based on normalized ratio to the β-actin protein level. ARI, aripiprazole; CLO, clozapine; CLO+MIF, clozapine with add-on MIF treatment; HAL, haloperidol; MIF, mifepristone; NC, normal control; RIS, risperidone; RIS+MIF, risperidone with add-on MIF treatment.

To verify whether the gene expressions of the six key regulators further caused changes in their protein expressions, the western blot experiments were also performed in the rats after antipsychotic treatment (Figure 3). Similarly, CLO and RIS caused significant reductions in the protein expression of PGRMC1 (Figure 3a) and INSIG-2 (Figure 3c), and significant elevations in the protein expressions of SCAP/SREBP (Figure 3d and f) as compared with NC. Again, none of the tested protein expressions were significantly affected by ARI or HAL (Figure 3a–f).

As there were no significant influences of SER on dietary intake and body weight (Supplementary Figure 1) as well as levels of lipid and hormone parameters (Table 1), the qPCR and western blot experiments were omitted from this SER group.

### Enhanced hepatic PGRMC1/INSIG-2 expressions by the add-on MIF treatment

In accordance with our data above, the *PGRMC1/INSIG-2* gene expressions were significantly reduced in both CLO and RIS groups (Figure 2c), whereas the *SCAP/SREBP* expressions were markedly increased (Figure 2d). Following the add-on MIF treatment, however, both reduced *PGRMC1/INSIG-2* and elevated *SCAP/SREBP* expressions appeared to be normalized without influencing *INSIG-1* expression (Figure 2c and d).

**Figure 4:**
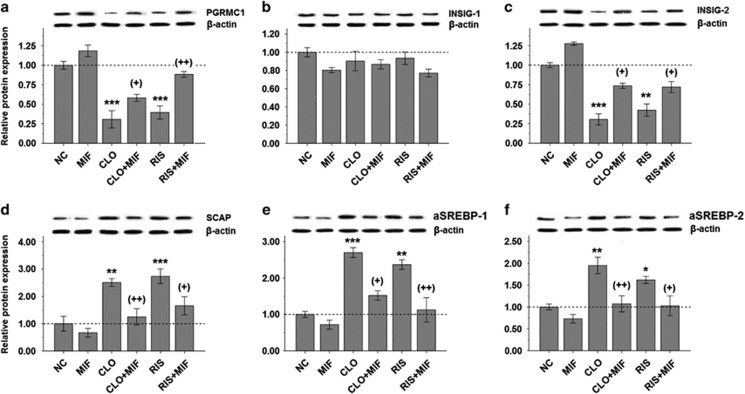
Comparisons of hepatic expression of PGRMC1/INSIG/SCAP/SREBP protein in CLO- or RIS-treated rats before and after the add-on MIF treatment using western blotting: (**a**) PGRMC1, (**b**) INSIG-1, (**c**) INSIG-2, (**d**) SCAP, (**e**) SREBP-1 and (**f**) SREBP-2. *P<0.05, **P<0.01 and ***P<0.0001 as compared with controls. +*P*<0.05 and ++*P*<0.01 in parentheses as compared with corresponding AAPD monotherapy group. Relative expression data were normalized from *β*-actin protein level. ARI, aripiprazole; CLO, clozapine; CLO+MIF, clozapine with add-on MIF treatment; HAL, haloperidol; MIF, mifepristone; NC, normal control; RIS, risperidone; RIS+MIF, risperidone with add-on MIF treatment.

Similar to gene expressions, the add-on treatment of MIF significantly reversed the effects of decreased PGRMC1/INSIG-2 (Figure 4a and c) and increased SCAP/SREBP (Figure 4d–f) protein expressions in either CLO or RIS groups. There were no significant changes of all tested protein expressions in the MIF treatment-alone group (Figure 4a–f).

## Discussion

### Effects of AAPDs and add-on MIF on weight gain and food intake

In the present study, there were no significant weight gain or increased food intake in male rats following 28-day treatment of all antipsychotic drugs tested (Supplementary Figure 1a and b) as well as an add-on treatment of MIF (Supplementary Figure 1c and d). In general, our findings were in accordance with those animal studies conducted in less than 1-month treatment with AAPDs such as olanzapine,^33^ CLO^34^ or other typical antipsychotic drugs.^35^ By contrast, the percentage of weight gain and accumulated food intake was significantly reduced following 21- and 28-day treatments of CLO (Supplementary Figure 1a and b). Similarly, Baptista *et al.*^36^ also demonstrated significant reduction in body weight and feeding in male rats after 21-day CLO treatment at a dose similar to our present study (20 mg kg^−1^); however, it is considered as a toxic effect. It is thus surmized that male rats appear to be more sensitive to the toxic effects of CLO than female rats.^36^

On the other hand, female instead of male rats were prone to AAPD-induced weight gain.^33, 35^ Moreover, rodents with the low doses of AAPD appear to be more effective in weight gain and food intake than those with high doses of AAPD. However, such low doses do not have any clinical relevancies.^37, 38^

In healthy men, however, there were significant weight gain or increased waist circumference within short-term (<1 month) treatment of AAPDs (for example, olanzapine or resperdone).^22, 23^ Similarly, SZ patients receiving AAPDs (specifically CLO and olanzapine) also showed significant problems with obesity and MetS.^39^ Such a discrepancy between human and animals may result from the differences in AAPD's dosage, food intake, age or gender effect.^34, 36^ Nevertheless, the present findings suggest significant elevations of metabolic parameters by AAPDs occurring before the presence of actual weight gain, which provides the temporal associations between AAPD medication and metabolic consequences. Together, our animal data support the notion that acute administration of AAPDs exerts a potent effect on metabolic regulation, which may contribute to the MetS seen in humans.^40^

### Overexpression of SCAP/SREBP by AAPDs

As illustrated in Figure 1a, the SREBPs are central in the allosteric control of a variety of lipid biosynthetic pathways.^6^ Previous studies by Fernø and colleagues^11, 12, 13, 14^ have demonstrated that AAPDs such as CLO, olanzapine or RIS markedly enhanced expressions of both SREBP-1 and SREBP-2 as well as their associated target genes regulating the *de novo* lipid and cholesterol synthesis in hepatocytes. In addition to the increased SREBP gene (Figure 2b) and protein (Figure 3e and f) expressions, our present data demonstrating increased lipid parameters (Table 1) lend further support that hepatic SREBPs were overexpressed in the CLO- or RIS-treated rats.^6, 11, 12^

The stearoyl-CoA desaturase (SCD1) is the rate-limiting enzyme that catalyzes the conversion of saturated long-chain fatty acids into monounsaturated fatty acids required for the *de novo* biosynthesis of triacylglycerol, phospholipids and cholesterol esters.^41^ It is a target of SREBP transcription factors and is directly involved in lipid homeostasis.^11^ A number of studies have found that different AAPD medications upregulate the expression of SREBP-controlled downstream lipogenic genes including *SCD1*.^11, 13, 42, 43^ They are in accordance with our present findings that CLO- or RIS-induced SREBP activation leads to increased lipid levels, which are mediated through the effects of SREBP on *SCD1* and other target genes. In the present study, we mainly focused on the upstream regulatory mechanisms of SREBP.

SCAP, a transport protein that escorts ia-SREBPs from the ER to Golgi apparatus (Figure 1a), is essential during the process of SREBP activation, and its activation could induce lipid synthesis in the liver.^44^ Figures 2b and 3d indicated that SREBP activation was accompanied by SCAP upregulation following the treatment of CLO or RIS. Our findings thus suggest that AAPD's influences already precede the process of their proteolytic maturation in the Golgi apparatus.

### Suppression of INSIG-2 gene and proteins by AAPDs

INSIG is a negative regulator of SCAP/SREBP.^7^ In response to the inhibition of INSIG, SCAP is released to escort SREBP to the Golgi apparatus for activation.^8^ In support of the previous findings that the *INSIG-2* gene may be associated with MetS in SZ patients following antipsychotics treatment,^9, 10^ our present data provide further evidence that the protein INSIG-2 rather than INSIG-1 was suppressed in the rat liver after treatment with either CLO or RIS (Figures 2a and 3b, c).

It is also known that the expression of INSIG-2 can be regulated by insulin in the liver.^45^ Contrary to our data showing significant reduction of INSIG-2 expression, levels of insulin were not altered significantly in the same CLO- or RIS-treated groups (Table 1). This paradox suggests that additional factor(s) may be accounted for the AAPD-induced reduction of INSIG expression.

### Role of PGRMC1 in AAPD-induced SREBP activation

As illustrated in Figure 1a, PGRMC1 is a progesterone receptor membrane component 1 that binds to INSIGs and SCAP. In the present data, the hepatic expression of PGRMC1 was downregulated in parallel with decreased INSIG-2 expressions (Figures 2a and 3a, c), which supported a role of PGRMC1 in the regulation of INSIGs during the CLO or RIS treatment.^16, 17, 18^

In addition to marked increases of lipid parameters, levels of serum progesterone and corticosterone were also increased in the CLO- and RIS-treated groups (Table 1), which may contribute to the suppression of PGRMC1 signaling.^20^

### Regulation of the PGRMC1 pathway by add-on MIF treatment

Cortisol is a glucocorticoid hormone, which can be metabolized to 11-hydroxyglucocorticoid by the 11β-hydroxysteroid dehydrogenase system.^46^ This enzyme system is abundantly present in the liver and adipose tissue, which is considered as an etiological factor linking to obesity and associated MetS.^47^ A previous study indicated that MIF, a potent glucocorticoid antagonist, was able to reverse the weight gain shown in young obese fa/fa Zucker rats.^48^ Several recent studies have further confirmed the effects of MIF on reducing or preventing AAPD-induced weight gain in healthy subjects^22, 23^ as well as in rats.^21^ However, the exact mechanism underlying the AAPD-induced weight gain remains unclear.

In the present study, our data demonstrate that both CLO and RIS significantly reduce the expression of PGRMC1 (Figures 2a and 3a) and, subsequently, lead to marked changes in INSIG/SCAP/SREBP pathways (Figures 2a, b and 3c–f). Following the add-on MIF treatment, however, such alterations in the PGRMC1 and associated INSIG/SCAP/SREBP pathways were reversed in both the CLO and RIS groups (Figures 2c, d and 4a, c–f). Thus, it is likely that the PGRMC1 pathway may be the therapeutic target for AAPD-induced weight gain (Figure 1b). Moreover, PGRMC1, INSIGs, SCAP and SREBPs are dynamically linked together in the AAPD-induced lipid biosynthesis as well as the anti-lipogenic action.

In keeping with the changes in the PGRMC1 expression, the add-on MIF also significantly reduced levels of CLO- or RIS-induced lipid parameters (Table 1). Previously, Takaishi *et al.*^49^ indicated that overexpression of hepatic INSIG-1 or -2 may be linked to the reduction of lipogenesis in obese Zucker diabetic fatty rats and in fasted/re-fed normal rats. Our data, however, indicated that expression of INSIG-2, not INSIG-1, was significantly reduced by either CLO or RIS antipsychotics (Figures 2a and 3b, c). Following add-on MIF, only INSIG-2 (Figures 2c and 4c) was upregulated, suggesting that PGRMC1/INSIG-2 may be the therapeutic target for the AAPD-induced cardiovascular risk over the long term.

Previously, Beebe *et al.*^21^ have demonstrated a significant weight gain in female Sprague–Dawley rats after a 35-day olanzapine treatment compared with vehicle controls. Following add-on MIF, however, a significant portion of olanzapine-induced weight gain disappeared, suggesting that MIF may mitigate weight gain by blocking the effects of glucocorticoid receptor.^22, 23^ In the present study, to avoid progesterone fluctuation during the menstrual cycle, only male rats were used. There were no apparent increases of weight gain or food intake in CLO, RIS, MIF-alone or MIF-add-on groups (Supplementary Figure 1c and d). Lacking weight gain is likely due to the gender difference. On the other hand, it is not clear why the CLO- or RIS-enhanced serum progesterone and corticosterone levels were not affected by the steroid receptor antagonist (Table 1).

In summary, our data demonstrated significantly increased hepatic expressions of SCAP/SREBP and the parallel inhibition of PGRMC1/INSIG-2, whereas levels of serum lipid and hormone parameters were markedly elevated in CLO- or RIS-treated rats. Such metabolic disturbances, however, can be modified by an add-on treatment of steroid antagonist MIF, suggesting that PGRMC1/INSIG-2 signaling may be a therapeutic target for AAPD-induced weight gain.

## Figures and Tables

**Table 1:** Effects of 4-week antipsychotic treatment and add-on mifepristone treatment on serum lipid and hormone parameters

*Experiments*	*Serum parameters*^a^	*Treatment groups (*n=*7, each)*					
		*NC*	*SER*	*HAL*	*ARI*	*RIS*^b^	*CLO*^b^
Antipsychotic treatment	Triacylglycerols	0.79±0.38	0.69±0.20	0.61±0.14	0.78±0.19	1.22±0.18*	1.21±0.30*
	Total cholesterol	1.50±0.32	1.57±0.30	1.53±0.36	1.82±0.33	2.07±0.52*	2.55±0.84**
	Free fatty acids	0.74±0.14	0.73±0.12	0.88±0.20	1.01±0.28	2.41±0.55***	2.94±0.85***
	Progesterone	44.02±28.89	124.58±11.04	80.11±48.81	65.37±25.95	221.97±81.53**	312.63±16.92***
	Corticosterone	0.54 ±0.26	0.12±0.11	0.82±0.40	0.88±0.29	3.14±1.15***	1.76±0.49**
	Insulin	36.66±12.14	39.24±11.88	38.24±11.82	32.01±11.24	40.96±8.46	42.96±12.13
							
		*NC*	*MIF*	*CLO*^b^	*CLO+MIF*^c^	*RIS*^b^	*RIS+MIF*^c^
Add-on MIF treatment	Triacylglycerols	0.55±0.33	0.41±0.15	1.86±0.56**	0.29±0.10^+++^	1.46±0.35**	0.29±0.07^+++^
	Total cholesterol	1.60±0.33	1.27±0.26	2.67±0.70**	1.34±0.26^++^	2.12±0.57*	1.03±0.25^+++^
	Free fatty acids	1.68±0.55	1.33±0.15	3.09±0.87*	1.31±0.48^+++^	2.62±0.54*	1.33±0.39^++^
	Progesterone	49.75±15.42	69.76±18.13	283.08±20.55**	234.18±84.23	139.71±73.97**	139.58±67.83
	Corticosterone	0.48±0.23	0.52±0.17	2.67±0.22***	2.13±1.21	1.82±0.47**	1.63±0.97
	Insulin	28.68±4.44	22.85±4.19	28.42±5.91	22.90±7.08	30.48±8.39	23.32±6.73

Abbreviations: ARI, aripiprazole; CLO, clozapine; HAL, haloperidol; MIF, mifepristone; NC, normal control; RIS, risperidone; SER, sertraline.

*P<0.05, **P<0.01 and ***P<0.0001 (for antipsychotic effects); ^++^*P*<0.01 and ^+++^*P*<0.0001 (for add-on MIF treatment effects).

a Serum triacylglycerol, total cholesterol and free fatty acid were expressed as mmol l^−1^, whereas progesterone, corticosterone and insulin were expressed as nmol l^−1^, μmol l^−1^ and mIU l^−1^, respectively.

b Significant differences were conducted between treatment-alone and NC groups.

c Significant differences were conducted between treatment-alone and add-on MIF treatment groups.
