# Supplementary material for: A potential mechanism underlying atypical antipsychotics-induced lipid disturbances
Source: Transl Psychiatry. 2015 Oct 20;5(10):e661–. doi: 10.1038/tp.2015.161 (PMC4930135; doi:10.1038/tp.2015.161)
Supplement: Supplementary Table 1 [file tp2015161x1.doc]

| **Supplementary Table 1** Experimental design of 4-week antipsychotic treatment and add-on mifepristone treatment. | | | | | | | |
| --- | --- | --- | --- | --- | --- | --- | --- |
|  |  |  |  |  |  |  |  |
|  | | | | | | | |
| *Experiment* | *Groups*  *(n=7 each)* | *Control* | *Antidepressant* | *Antipsychotics* | | | |
|  |  |  |  | *Typical* | *Atypical* | | |
|  |  | *Vehicle^a^* | *Sertraline (7 mg/kg/day)^a^* | *Haloperidol (1 mg/kg/day)^a^* | *Aripiprazole (2 mg/kg/day)^a^* | *Risperidone (1 mg/kg/day)^a^* | *Clozapine (21 mg/kg/day)^a^* |
|  |  |  |  |  |  |  |  |
| Antipsychotic treatment | NC | + | - | - | - | - | - |
|  | SER | - | + | - | - | - | - |
|  | HAL | - | - | + | - | - | - |
|  | ARI | - | - | - | + | - | - |
|  | RIS | - | - | - | - | + | - |
|  | CLO | - | - | - | - | - | + |
|  |  | *Control* | *Atypical antipsychotics* | | *Steroid antagonist* |  |  |
|  |  | *Vehicle* | *Clozapine (21 mg/kg/day)^a^* | *Risperidone (1 mg/kg/day)^a^* | *Mifepristone (200 mg/kg/day)^b^* |  |  |
|  |  |  |  |  |  |  |  |
| Add-on MIF treatment | NC | + | - | - | - |  |  |
|  | MIF | - | - | - | + |  |  |
|  | CLO | - | + | - | - |  |  |
|  | CLO+MIF | - | + | - | + |  |  |
|  | RIS | - | - | + | - |  |  |
|  | RIS+MIF | - | - | + | + |  |  |

Abbreviations: NC, normal control; SER, sertraline; HAL, haloperidol; ARI, aripiprazole; RIS, risperidone; CLO, clozapine; MIF, mifepristone.

^a^ Intraperitoneal injection of 0.9% saline containing 0.5% Tween 80 (vehicle) or designated drug.

^b^ Add-on MIF treatment was administrated by oral gavage daily.
