# Supplementary material for: A potential mechanism underlying atypical antipsychotics-induced lipid disturbances
Source: Transl Psychiatry. 2015 Oct 20;5(10):e661–. doi: 10.1038/tp.2015.161 (PMC4930135; doi:10.1038/tp.2015.161)
Supplement: Supplementary Table 2 [file tp2015161x2.doc]

| **Supplemental Table 2**  Primer sequences used for the qPCR analysis | | | | |
| --- | --- | --- | --- | --- |
| **Gene** | **Forward primer (5’→3’)** | **Reverse primer (5’→3’)** | **Accession number** | **Amplicon length** |
| PGRMC1 | AGGGACCATACGGGGTCTT | AGCAGTTTTCCCCACGTGAT | NM021766.1 | 190 bp |
| INSIG-1 | TCACCTGGGAGAACCACACA | CGATCAAACGTCCACCACAAGC | NM022392.1 | 171 bp |
| INSIG-2 | AGCCTCAGCTGTGATTGGAC | CTTTAGCACTGGCGTGATTT | NM178091.4 | 137 bp |
| SCAP | TGAGCCTAAAACCCTACAG | AAACTTGGCATGGTAGCG | NM001100966.1 | 148 bp |
| SREBP-1 | CGGCGCTCTTGACCGACAT | AGCTCTCAGGAGAGCTGGCACC | NM001276707.1 | 147 bp |
| SREBP-2 | AGGCTCTGGCCGCAATGTAC | AGCTTCACGAAGACGCTCA | NM001033694.1 | 123 bp |
| β-Actin | CATCCTGCGTCTGGACCTGG | TAATGTCACGCACGATTTCC | NM031144 | 116 bp |
